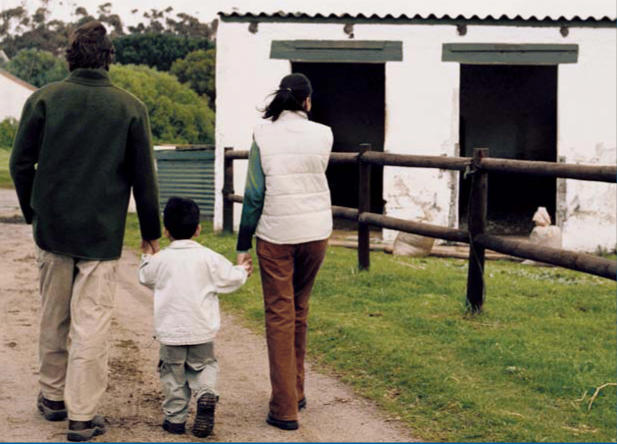# Headliners: Pesticides: Neurobehavioral Deficits in Children from Agricultural Communities

**Published:** 2006-03

**Authors:** Jerry Phelps

Rohlman DS, Arcury TA, Quandt SA, Lasarev M, Rothlein J, Travers R, et al. 2005. Neuro-behavioral performance in preschool children from agricultural and non-agricultural communities in Oregon and North Carolina. Neurotoxicology 26:589–598.

Most research on the neurobehavioral effects of organophosphate (OP) pesticides has focused on adult occupational exposures. However, the developing organ systems of children can be especially sensitive to these chemicals. Now NIEHS-supported scientists Linda McCauley of the University of Pennsylvania, Thomas A. Arcury of Wake Forest University, and Joan Rothlein of Oregon Health & Science University, with their colleagues, report modest differences in neurobehavioral perfomance between young children from agricultural communities and those from nonagricultural communities.

Research has shown that children may be chronically exposed to low doses of pesticides that do not cause symptoms evident in routine examinations. These exposures can result from hand-to-mouth behavior and more time spent on the floors of their homes and in contact with soils. They can also occur through food, drinking water, and indoor and outdoor use of pesticides. In general, children of agricultural workers are at special risk of pesticide exposure because their homes are usually close to fields where application occurs, and they can encounter take-home exposure on parents’ clothing.

The researchers recruited children of Latino immigrants. All of the children recruited were aged 48 to 71 months. At least one parent of each child from the agricultural communities worked in agriculture at the time of the study. Neither parent of children from nonagricultural communities had worked in agriculture in the past year.

The researchers used a battery of behavioral tests to measure the children’s cognitive and neurobehavioral function. Eleven of the measures showed no significant deficit between the two groups. However, the agricultural children did perform significantly worse on two tests: finger tapping (which measures response speed) and a test for visual memory. The results are consistent with effects seen in previous research on adults with documented low-level exposure to OP pesticides.

This study points out the need for additional larger studies aimed at determining whether low-level OP pesticide exposures produce deficits in standardized test performance among children of agricultural workers. It also illustrates the importance of proper pesticide application and good hygiene in pesticide applicators to prevent exposures in their children.

## Figures and Tables

**Figure:**